# Interaction of Severe Acute Respiratory Syndrome Coronavirus 2 and Diabetes

**DOI:** 10.3389/fendo.2021.731974

**Published:** 2021-10-06

**Authors:** Shiying Shao, Qin Yang, Ruping Pan, Xuefeng Yu, Yong Chen

**Affiliations:** ^1^ Division of Endocrinology, Tongji Hospital, Huazhong University of Science and Technology, Wuhan, China; ^2^ Branch of National Clinical Research Center for Metabolic Diseases, Hubei, China; ^3^ Division of Pathology, Tongji Hospital, Huazhong University of Science and Technology, Wuhan, China; ^4^ Department of Nuclear Medicine, Tongji Hospital, Huazhong University of Science and Technology, Wuhan, China

**Keywords:** SARS-CoV-2 (2019-nCoV), COVID – 19, diabetes - quality of life, OADs, immunocellular response

## Abstract

Severe acute respiratory syndrome coronavirus 2 (SARS-CoV-2) is causing a worldwide epidemic. It spreads very fast and hits people of all ages, especially patients with underlying diseases such as diabetes. In this review, we focus on the influences of diabetes on the outcome of SARS-CoV-2 infection and the involved mechanisms including lung dysfunction, immune disorder, abnormal expression of angiotensin-converting enzyme 2 (ACE2), overactivation of mechanistic target of rapamycin (mTOR) signaling pathway, and increased furin level. On the other hand, SARS-CoV-2 may trigger the development of diabetes. It causes the damage of pancreatic β cells, which is probably mediated by ACE2 protein in the islets. Furthermore, SARS-CoV-2 may aggravate insulin resistance through attacking other metabolic organs. Of note, certain anti-diabetic drugs (OADs), such as peroxisome proliferator-activated receptor *γ* (PPARγ) activator and glucagon-like peptide 1 receptor (GLP-1R) agonist, have been shown to upregulate ACE2 in animal models, which may increase the risk of SARS-CoV-2 infection. However, Metformin, as a first-line medicine for the treatment of type 2 diabetes mellitus (T2DM), may be a potential drug benefiting diabetic patients with SARS-CoV-2 infection, probably *via* a suppression of mTOR signaling together with its anti-inflammatory and anti-fibrosis function in lung. Remarkably, another kind of OADs, dipeptidyl Peptidase 4 (DPP4) inhibitor, may also exert beneficial effects in this respect, probably *via* a prevention of SARS-CoV-2 binding to cells. Thus, it is of significant to identify appropriate OADs for the treatment of diabetes in the context of SARS-CoV-2 infections.

## 1 Introduction

Coronavirus disease 2019 (COVID-19), caused by severe acute respiratory syndrome coronavirus 2 (SARS-CoV-2)-a novel β-coronavirus, has influenced over 100 million people. This SARS-CoV-2 strain has become the third most lethal pathogenic human coronavirus since SARS-CoV-1, which was responsible for severe acute respiratory syndrome (SARS) in 2002 (1), and Middle-East respiratory syndrome coronavirus (MERS-CoV), which was responsible for MERS in 2012 (2).

The coronavirus belongs to a large family of single-stranded, enveloped RNA viruses, which can be divided into four genera: α-, β-, γ- and δ- coronavirus (3). All of the above 3 viruses belong to the β-genus (1, 2, 4), sharing a structural analogy (4, 5). Although the pathophysiological mechanisms and clinical invasiveness of SARS-CoV-2 have not yet been fully investigated, it probably, at least in part resembles SARS-CoV-1 and MERS-CoV.

There are more and more studies demonstrating the association between COVID-19 and diabetes, disclosing that these two diseases appear to be bi-directional. Diabetes may magnify the pathogenicity of SARS-CoV-2 because part of pathological mechanisms of diabetes overlaps with COVID-19, resulting in an increase in susceptibility and severity of COVID-19 among diabetic patients. Investigation of the underlying mechanisms would contribute to improve the clinic outcomes of these patients. On the other hand, SARS-CoV-2 infection may induce new onset diabetes, which could be possibly overlooked by nonendocrinologists. Thus, this review focuses on the bi-directional interaction between SARS-CoV-2 and diabetes and the possible mechanisms. Moreover, some glucose lowering agents may provide extra benefits for COVID-19 treatment, which may disclose some new clues for the treatment of non-diabetic patients with SARS-CoV-2 infection.

## 2 Impact of Pre-Existing Diabetes on COVID-19 Progression

Emerging data indicates that patients with diabetes are at a higher risk of SARS-CoV-2 infection. In one SARS-CoV-2 study, which includes 1,099 patients from 552 hospitals in China, the rate of diabetes was around 15%, including children and adults (6). Diabetes was reported in 34.6% of the subjects with a composite endpoint including intensive care unit admission, requirement for ventilation, and death (7). Moreover, a study with 5700 patients in New York City, USA, identified that one of the most common comorbidities caused by SARS-CoV-2 was diabetes, with a rate of 33.8% (8). Stokes et al. reported that, as of May 30, 2020, in a population of about 1.3 million patients with SARS-CoV-2 infection in the USA, around 30% of whom have been suffering from diabetes (9). Of note, the exact rates of type 2 diabetes mellitus (T2DM) in patients with SARS-CoV-2 infection vary. The inconsistent rate can be attributed to the diagnosis of both conditions, which depends on the testing methods of SARS-CoV-2 (nasal test or pharyngeal test, nucleic acids or antibodies), enrolled population (out-patient or hospitalized patients), the severity of illness (mild or severe symptom), sample sizes, and different locations.

In addition, diabetic patients are associated with a more severe course of COVID-19, who account for a higher proportion of intensive care unit (ICU)-admitted cases. A retrospective cohort study of 7,337 patients with SARS-CoV-2 infection in China showed that subjects with T2DM had a significantly higher mortality and morbidity of multiple organ injury than the non-diabetic individuals (10). The authors further demonstrated that the in-hospital death rate was significantly lower in the well-controlled blood glucose group relative to the poorly controlled group (10). An analysis of a randomly selected subset of fatal SARS-CoV-2 cases in Italy revealed a high prevalence of diabetes, with a rate as high as 35.5% (11). A COVID-19 study in Pisa, Italy identified that the mortality of SARS-CoV-2 patients was greater in hyperglycemia subjects (about 39.4%) than in patients with normal glycemia (about 16.8%) (12). The authors concluded that hyperglycemia acts as an independent factor associated with severe prognosis in hospitalized patients with SARS-CoV-2 infection (12). Lately, a large study relevant to diabetic patients from the Chinese Center for Disease Control and Prevention with 72,314 SARS-CoV-2 cases across the Mainland China also showed a higher fatality rate in diabetic patients (7.3% vs. 2.3% overall) than regular patients, second only to patients with cardiovascular diseases (13). A meta-analysis based on six published clinical studies further confirmed the negative influence of diabetes on SARS-CoV-2 infection, that diabetic patients displayed 2.95-fold higher risk of fatality after SARS-CoV-2 infection, compared to those without diabetes (14). However, most of these studies did not indicate whether the included patients were type 1 diabetes mellitus (T1DM) or T2DM. In addition, stratified analyses according to Hemoglobin A1c (HbA1c), insulin sensitivity, β cell function, and c peptide level were limited.

Likewise, patients with SARS-CoV-2 infection display increased risk of ketosis and hyperglycaemic emergencies. One observation retrieved from the Chinese population described that 42 (6.4%) out of 658 patients with SARS-CoV-2 infection presented with ketosis on admission with no obvious fever or diarrhea, and 3 (20.0%) out of the 15 COVID-19 cases with diabetic ketosis developed acidosis (15). Of note, there was only one T1DM among these included patients, indicating that it was T2DM rather than T1DM tended to develop ketosis under the context of COVID-19. A recent retrospective analysis characterized 35 T1DM and T2DM patients with hyperglycaemic emergencies including diabetic ketoacidosis (DKA), hyperosmolar hyperglycaemic state (HHS), and hyperglycaemic ketosis in the context of SARS-CoV-2 from three hospitals in north London in UK. They concluded that SARS-CoV-2 is associated with the occurrence of hyperglycemia emergencies with overrepresentation of T2DM in patients presenting with DKA and long-lasting ketosis (16). Accordingly, ketone monitoring becomes very important among diabetic patients in the COVID-19 epidemics, especially in T2DM patients. Howbeit, the sample size of these studies was relatively small. Larger study population and longer observation were needed.

## 3 Potential Mechanisms of Diabetes on COVID-19 Progression

The reasons for increased risk of coronaviral infection in diabetic patients are not fully identified. Multifarious mechanisms are possibly involved in the disease susceptibility and progression. Particularly, previous investigations on SARS-CoV-1 and MERS-CoV may provide evidence on the pathophysiology of SARS-CoV-2 infection in patients with diabetes.

### 3.1 Diabetes and Lung Dysfunction

Diabetes is associated with a change in lung physiology and structure. It has been reported an increase of extracellular matrix synthesis in the pulmonary alveolar wall in diabetic rat model, resulting in a reduction of lung elasticity and a decrease in alveolar space (17). Furthermore, Weynand et al. identified that alveolar epithelial basal lamina in diabetic patients was significantly thicker than that in healthy subjects. This result reveals that hyperglycemia leads to the thickening of the pulmonary microvascular wall (18), which potentially participates in the damage of alveolar-capillary membrane, and in turn, reduces the gas diffusion rate in diabetic patients (19).

The above structure changes in lung result in abnormalities of pulmonary function including lung volume, pulmonary diffusing capacity, pulmonary ventilation, bronchomotor tone, and neuroadrenergic bronchial innervation (20). A prospective study of 125 patients with T2DM revealed that absolute and percentage-predicted lung function measures including forced vital capacity (FVC), forced expiratory volume in 1 s (FEV1), vital capacity (VC), and peak expiratory flow (PEF) were significantly reduced after 7-year follow-up (21).

**Figure 1:**
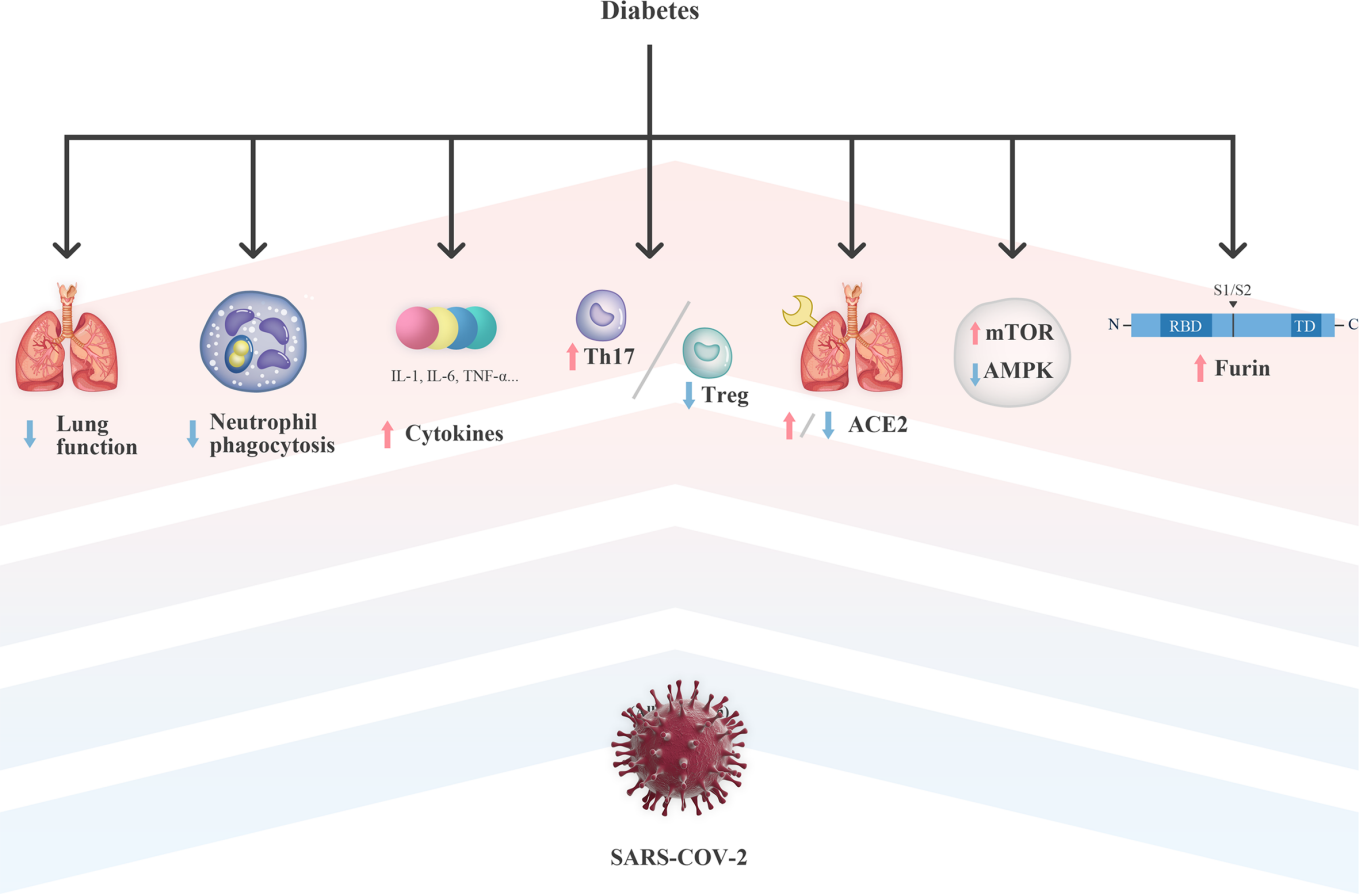
Potential mechanism of diabetes on the susceptibility and severity of COVID-19. ACE2, Angiotensin-converting enzyme 2; AMPK, Adenosine 5’-monophosphate-activated protein kinase; IL, interleukin; mTOR, mechanistic target of rapamycin; RBD, receptor binding domain; SARS-CoV-2, severe acute respiratory syndrome coronavirus 2; TD, transmembrane domain; Th17, T helper 17; TNF-α, tumor necrosis factor-α; Treg, regulatory T.

Accordingly, the impaired pulmonary function in patients with pre-existing diabetes may partly explain the susceptibility and poor outcomes of diabetic patients after SARS-CoV-2 infection (**Figure 1**).

### 3.2 Diabetes and Immune Dysfunction

Diabetes, even a short-term hyperglycemia, has been shown to impair the balance of immune system (22). Although the immunologic mechanisms induced by SARS-CoV-2 are not fully elucidated, existing data derived from the close counterparts SARS-CoV and MERS-CoV may bring some enlightenment. Part of mechanisms of immune dysfunction between diabetes and COVID-19 may cross, including impaired neutrophil function, pro-inflammatory inclination and T cell imbalance under hyperglycemia.

#### 3.2.1 Diabetes and Impaired Neutrophil Function

In the innate immune response, neutrophils play a crucial role in chemotaxis and phagocytosis. Under hyperglycemia, neutrophil dysfunction has been observed in both humans and rodents (23). Possibly, acute hyperglycemia impairs the respiratory burst capacity of neutrophils, the process by which immune cells release toxic chemicals and kill pathogensr (24). Moreover, hyperglycemia reduced neutrophil degranulation (25) and impaired superoxide production from activated neutrophils (26).

Thus, impaired neutrophil function leads to an increase in risk and severity of infections (**Figure 1**), suggesting that glucose homeostasis in patients with SARS-CoV-2 infection may help to stabilize the ability of neutrophils for a proper innate immune response. Interestingly, a significant increase in neutrophil counts was observed in patients with both diabetes and SARS-CoV-2 infection (10, 12). Howbeit, few literature have described the function of neutrophils under the context of SARS-CoV-2 infection, which requires further investigation.

#### 3.2.2 Diabetes and Pro-Inflammatory State

It has been known that both diabetes and obesity cause a low grade pro-inflammatory state in the body with increased secretion of cytokines including interleukin (IL)-1, IL-6, IL-8 and tumor necrosis factor-α (TNF-α) (23). In diabetic and obese patients, excess cytokines in circulation keep the immune system in “threat” mode.

This hypercytokinemia seems to play a crucial role in the development of pulmonary fibrosis (27, 28). Evidences show that cytokine storm, also known as cytokine release syndrome (CRS), might be involved in lung injury and increased mortality, thus playing a major role in severe COVID-19 cases. Inflammatory cytokines and chemokines, including IL-1β, IL-2, IL-4, IL-6, IL-7, IL-10, IL-12, IL-17, TNF-α, interferon- γ (IFNγ), interferon gamma-induced protein 10 (IP10) and monocyte chemoattractant protein-1 (MCP-1), were significantly elevated in COVID-19 patients (29–34), some of which overlap with the cytokine panel in diabetes.

Among those overlapped cytokines, IL-6 is of particular interest and appears to be closely related to the occurrence of severe SARS-CoV-2 infection. It is an important pleiotropic cytokine, which is involved in acute inflammatory response and lung injury (35). In addition, elevated levels of IL-6 were observed in patients with SARS in 2004 and, in particular, were positively correlated to disease severity (36). A retrospective, multicentre cohort study in Wuhan in this epidemic found that IL-6 levels were closely associated with the mortality in patients with SARS-CoV-2 infection (34). Likewise, ICU patients with severe pneumonia in another study also showed a significant elevation in plasma level of IL-6 (37).

Importantly, serum levels of IL-6 in patients with diabetes and SARS-CoV-2 infection were significantly higher than those in non-diabetic patients (10, 38). This might be explained by that the increased cytokine baseline level and pro-inflammatory state observed in diabetic patients are further amplified by SARS-CoV-2 infection (**Figure 1**), resulting in an aggressive inflammatory response, even CRS.

#### 3.2.3 Diabetes and T Cell Imbalance

It has been demonstrated that in patients with either T1DM or T2DM, some “helpful” immune cells including certain subsets of CD4^+^ and CD8^+^ T cells are decreased, while some proinflammatory immune cells, such as T helper 17 (Th17) cells, are increased (39). Data supports the concept that Th17/regulatory T (Treg) cell mediated immune responses are involved in the pathogenesis in obesity-related T2DM (39).

A previous evidence demonstrated that acute intracellular bacterial infection in the diabetic host was associated with the Th17/Treg-mediated immune imbalance, resulting in exaggerated inflammatory cascades (40). In 2019, Kulcsar et al. created a diabetic mouse model with the expression of human dipeptidyl peptidase 4 (DPP4), which is identified as the cellular binding site for MERS-CoV (41). These mice developed severe infection and displayed a more prominent Th17 response with increased levels of IL-17. Due to the viral homology between SARS-CoV-2 and MERS-CoV, it is speculated that Th17/Treg-mediated immune responses may play an essential role in the aggravation of SARS-CoV-2 infection in diabetic patients.

It has been revealed that the pathological characteristics of COVID-19 observed by postmortem pathology greatly resemble those observed in SARS-CoV-1 and MERS-CoV infection (42). In this SARS-CoV-2 case, pro-inflammatory T cells were overactivated, manifested by increase of CCR4^+^CCR6^+^ Th17 cells and high cytotoxicity of CD8^+^ T cells (42). Notably, an extensive multi-omics dataset demonstrated that the frequencies of CD4^+^ T cells that can secrete Th17 cytokines, including IL17-A and IL17-F, are increased in SARS-CoV-2 infected patients (43). Moreover, Pacha et al. indicated that the severity of disease is positively correlated with plasma levels of IL-17 and other Th17 cell-related cytokines (33). Specifically, Qin et al. identified that patients with SARS-CoV-2 infection also show lower levels of Treg cells, which are even lower in severe cases (44).

Thus, SARS-CoV-2 may affect circulating immune cells and exacerbate the uncoordination of the innate immune system, which are previously present in diabetic patients, and therefore, resulting in a deluge of inflammatory cytokines and a further damage of the organs (**Figure 1**).

### 3.3 Diabetes and mTOR Signaling

During the progression of T2DM, Adenosine 5’-monophosphate (AMP)-activated protein kinase (AMPK) is inactivated, leading to a chronic overactivation of mechanistic target of rapamycin (mTOR) C1 (45), which has been associated with insulin resistance and progression of diabetes-induced complications (46).

Increasing evidences also highlight mTOR as a key factor in regulating the replication of viruses. Rapamycin, which is a mTOR inhibitor, was found to be a potent inhibitor for the RNA replication of hepatitis C virus (HCV) (47) and MERS-CoV (48). In patients with severe H1N1 pneumonia, early adjuvant treatment with corticosteroids and mTOR inhibitor effectively blocked the expression of viral protein and the release of virion, and therefore, significantly improved the prognosis of disease (49). These findings disclose a potential anti-viral treatment for the patients with SARS-CoV-2 infection through an inhibition of the metabolic sensor mTOR.

It is known that stress-induced Regulated in Development and DNA Damage Responses 1 (REDD1) negatively regulates mTOR activity (50). Interestingly, IL-6, which is closely related to the progression and severity of COVID-19, directly enhances the activation of mTOR by reducing the expression of REDD1 (50). On the contrary, IL-37 performs its immunosuppressive activity by targeting mTOR as well, suggesting that IL-37 administration may be a possible therapeutic strategy for SARS-CoV-2 treatment (51). More importantly, a recent study tested 16 repurposable anti-human coronavirus (HCoV) drugs, that may provide a synergistic effect for the treatment of SARS-CoV-2, by analyzing their drug-gene signatures and HCoV-induced transcriptomics in human cell lines. Among these candidates, Sirolimus, which consists of an mTOR inhibitor and dactinomycin, may be a potential drug for COVID-19 treatment (52).

Thus, these findings suggest that the dysregulation of AMPK/mTOR signaling in the setting of T2DM may be a plausible explanation for the increased susceptibility of diabetic patients to COVID-19 (**Figure 1**).

### 3.4 Diabetes and ACE2

Angiotensin-converting enzyme 2 (ACE2) is a transmembrane glycoprotein which is expressed in organs throughout the whole human body, mainly in lung, intestine, kidney, blood vessels, and pancreas. The renin-angiotensin system (RAS) signaling pathway comprises both ACE, which metabolizes angiotensin I (Ang I) into angiotensin II (Ang II), and ACE2, which degrades Ang II to Ang 1-7 peptide (53). It has been reported that both SARS-CoV-1 and SARS-CoV-2 bind and gain entry to infected cells through ACE2 (4, 54), so that increased expression of ACE2 may contribute to increased chances of SARS-CoV-2 infection.

However, the role of ACE2 in the crosstalk between COVID-19 and diabetes is still a matter of debate, which may be largely attributed to the inconsistent expression of ACE2 in different tissues and stages of diabetes (**Figure 1**). The effect of hyperglycemia on the expression of ACE2 in different organs was investigated in non-obese diabetes (NOD) mouse models with the increased expression of ACE2 in the serum, liver, and pancreas (55). In rats with streptozotocin-induced diabetes, there also showed an upregulation of ACE2 in isolated jejunal enterocytes (56). In addition, in adipocytes from obese and diabetic patients, an increased expression of ACE2 was also observed, suggesting that adipose tissue is a viral reservoir and could be a potential target for antiviral treatment (57). Recently, a phenome-wide Mendelian randomization study has explored and identified T2DM as a disease causally associated with increased expression of ACE2 (58).

Surprisingly, Roca-Ho et al. has reported that the activity ratio of ACE2/ACE in the lung was significantly decreased in the late-stage of diabetes in NOD Mouse (55). Although a study from Wysocki et al. has concluded that ACE2 expression was increased at the posttranscriptional level in the renal cortex in diabetic mice (59), ACE2 expression was shown to be decreased in the tubules in individuals with overt diabetic nephropathy (60).

Remarkably, it remains controversial whether an increased expression of ACE2 is responsible for the increased infectivity and severity of COVID-19. Some authors even consider that ACE2 plays a beneficial role in patients with COVID-19 (61, 62). It was demonstrated that ACE2 protects murine lungs from acute respiratory distress syndrome (ARDS) by decreasing inflammation and vascular permeability (63, 64). Previous studies have shown that the transmembrane spike (S) glycoprotein of SARS-CoV-1 downregulates ACE2 by shedding its ectodomain, an enzymatically active domain, and transforming it to soluble ACE2 (sACE2) (65–68). The biological function of sACE2 remains poorly investigated. However, it is assumed that sACE2 may act as a competitive interceptor for SARS-CoV-2 by inhibiting the binding of the S protein to ACE2 (69). A recent study has shown that treatment with clinical-grade human recombinant soluble ACE2 (hrsACE2) *in vitro* significantly inhibited the growth of SARS-CoV-2 in the monkey kidney cells (70), indicating that sACE2 potentially prevents SARS-CoV-2 infection. Howbeit, whether sACE2 plays a role in the disease progress in diabetic patients with SARS-CoV-2 infection remains unknown.

Taken together, ACE2 not only serves as a portal entry for SARS-CoV-2, but also plays a protective role against lung injury in its soluble form. The contradictory function of ACE2, especially, its role in the state with both diabetes and SARS-CoV-2 requires further investigations.

### 3.5 Diabetes and Furin

Afore mentioned transmembrane S protein of coronavirus is composed of two functional subunits: S1 region, which is responsible for its binding to the host cell ACE2 receptor, and S2 region, which is responsible for fusion of the viral RNA and cellular membranes. The S1 region could be cut and released by proteases, following by the entry of virus into the cells (71). It was previously discovered that MERS-CoV S protein can be activated by furin, a common protease responsible for membrane fusion (72). Likewise, it has been identified a furin cleavage site at the S1/S2 boundary of SARS-CoV-2 (73), indicating that furin could also cleave SARS-CoV-2 S protein (74) and possibly promote the viral entry.

Interestingly, diabetic patients show increased levels of furin (75). It may be driven by osteopontin, which is a cytokine-like matrix-associated phosphoglycoprotein and is shown to be elevated in diabetes (76). In addition, both T1DM and T2DM are associated with higher levels of plasmin(ogen), a protease enzyme that can cleave S protein of SARS-CoV-2 at furin site (77, 78). Thus, increased levels of furin and plasmin may aggravate the viral infection through assisting their entry, fusion, duplication, and release in cells.

## 4 COVID-19 May Trigger/Worsen the Development of Diabetes

As described above, diabetes worsens SARS-CoV-2 infection through mechanisms including impairing the pulmonary structure and function, disturbing the immune function, enhancing the expression of ACE2, overactivating the mTOR signaling, and inducing the furin levels. However, interplay between diabetes and COVID-19 appears to be bi-directional, as new onset diabetes has been observed in patients with SARS-CoV-2 infection (79). Multicenter regional data from North West London reported that thirty children, aged 23 months to 16.8 years, displayed new onset T1DM in this season. In comparison with the data from a typical year, it represents an additional 12-15 new T1DM cases with an increase rate of 80% during the COVID-19 pandemic (80).

These observations revealed a potential diabetogenic effect of COVID-19. The so induced diabetes may be different from the well-recognized notion of hyperglycemia induced by severe illness-associated stress response. To address the issues about the frequency, phenotype, and pathophysiology of SARS-CoV-2- induced new onset diabetes, an international group of leading diabetes researchers participating in the CoviDIAB Project have established a global registry of patients with SARS-CoV-2-related diabetes (covidiab.e-dendrite.com) (81).

The theoretical basis of new onset diabetes induced by SARS-CoV-2 could be supported by the findings of ACE2 expression in both exocrine and endocrine pancreas (islet cells) (82). In addition to lung, SARS-CoV-2 also attacks other organs and tissues including liver, brain, cardiovascular system, gastrointestinal system and pancreas, largely because of the wide distribution of ACE2 in those organs. Interestingly, the expression of ACE2 on the mRNA level in pancreas is slightly higher than that in the lung (82). Thus, SARS-CoV-2 probably causes the damage of pancreatic islets *via* ACE2 in pancreas (**Table 1**).

**Table 1:** Potential mechanisms of SARS-CoV-2 that triggers and worsens the development of diabetes.

Potential mechanism	Reference
SARS-CoV-2 causes the direct damage of pancreatic islets, partly *via* ACE2 in pancreas	(82, 83)
SARS-CoV-2 causes abnormal immune response such as inflammatory storm and T cell imbalance	(84)
SARS-CoV-2 worsens the insulin resistance through attacking key metabolic organs such as the liver, adipose tissue, and the small intestine	(55–57)
Medications for SARS-CoV-2 treatment, such as glucocorticoid and HIV PIs, impairs insulin sensitivity	(85, 86)

ACE2, Angiotensin-converting enzyme 2; HIV, Human immunodeficiency virus; PI, protease inhibitor; SARS-CoV-2, severe acute respiratory syndrome coronavirus 2; T2DM, type 2 diabetes mellitus.

SARS-CoV-2 infection may not only worsen the preexisting diabetes but also cause new cases of diabetes in non-diabetic subjects through a direct pancreatic damage and a resultant impairment of insulin secretion from β-cells. This can be partly proved by the study from Wang and colleagues (83). It was found that 9 out of 52 hospitalized patients with SARS-CoV-2-associated pneumonia in China displayed a pancreatic injury, which was determined by the plasma levels of amylase and lipase. Of note, 6/9 of these subjects exhibited moderate increases in plasma glucose (83). This phenomenon is in accordance with what is observed in patients after SARS-CoV-1 infection at the beginning of this century. Yang et al. reported that patients with SARS-CoV-1 infection, who had never received glucocorticoids, presented significantly higher levels of fasting plasma glucose compared to patients with non-SARS pneumonia (87). They supposed that SARS-CoV-1 may mediate the damage of pancreatic β-cells and resulted in a development of ‘acute diabetes’ in patients with SARS (87). In fact, SARS-CoV-1 has been identified in the pancreas of the patients who died of SARS by means of immunohistochemistry and *in situ* hybridization (88). Thus, coronavirus, SARS-CoV-1 as well as SARS-CoV-2, could be one of the pathogens and trigger the development of diabetes.

Apart from direct pancreatic β-cell damage, abnormal immune response such as inflammatory storm and T cell imbalance could also be another explanation for the new onset diabetes caused by SARS-CoV-2 (**Table 1**). As described previously, cytokines including IL-1β, IL-6, IL-17, TNF-α, and IFNγ were significantly elevated in patients with SARS-CoV-2 infection (29–34). Interestingly, the prospective population-based European Prospective Investigation into Cancer and Nutrition (EPIC)-Potsdam study revealed that a combined elevation of IL-1β and IL-6 has been shown to independently increase the risk of the development of T2DM (84). Th17 cytokine profile, which plays a major role in the regulation of inflammation and hyperglycemia, could mathematically predict T2DM in obese people (89). These findings further suggest that SARS-CoV-2 might trigger the development of new onset diabetes through an overactivation of the immune system.

Moreover, SARS-CoV-2 could worsen the insulin resistance in patients with pre-existing T2DM. As ACE2 expression is particularly amplified in key metabolic organs such as the liver, adipose tissue, and the small intestine (55–57), SARS-CoV-2 may attack these organs, resulting in insulin insensitivity and an exacerbation of hyperglycemia. In addition, it has been reported that acute viral infection by murine cytomegalovirus (MCMV) could induce a rapid development of transient insulin resistance through a specifical downregulation of the insulin receptors in skeletal muscle in rodents (90). Whether SARS-CoV-2 enhances the progression of systemic insulin resistance and its mechanisms still require further investigations.

Notably, glucocorticoid, which is an essential medication to control CRS in COVID-19, impairs insulin sensitivity and result in hyperglycemia too. Human immunodeficiency virus (HIV) protease inhibitors (PIs), such as Lopinavir-ritonavir, are used for the treatment of COVID-19. However, it is reported that HIV PIs may acutely and reversibly inhibit the insulin-responsive glucose transporter 4, leading to peripheral insulin resistance and impaired glucose tolerance (85). In a cross-sectional study recruited 710 HIV-infected patients, lopinavir/ritonavir was found to be significantly associated with some metabolic syndromes after adjustment for age and BMI (86). Thus, some medications, such as glucocorticoid and HIV PIs, which are used for SARS-CoV-2 treatment, should be further evaluated, particularly in SARS-CoV-2 infected patients with pre-existing diabetes or at high risk of diabetes (**Table 1**).

## 5 Treatment of COVID-19 in Patients With Diabetes

In concerning to an optimized treatment of patients with coexistent diabetes and COVID-19, a glycemic control seems to be very urgent (10, 91). However, there are still several cautions need to be taken to achieve the appropriate therapeutic strategies, especially in T2DM patients.

### 5.1 Metformin

As mentioned above, SARS-CoV-2 infection might worsen the prognosis of diabetes by dysregulating the AMPK/mTOR signaling (**Figure 1**) (45, 46, 51, 52). Observations suggest that activating AMPK and/or inhibiting mTOR-mediated signaling could be novel therapeutic intervention strategies for COVID-19 treatment (51, 52). Of note, dimethylbiguanide metformin is so far a first-line medication for the treatment of T2DM, which works as an AMPK activator (92). To a certain extent, this molecular mechanism makes the classical medication possible as antivirals in patients with COVID-19.

Interestingly, although a hypoglycemic effect of metformin was discovered in the 1920s, it was disregarded until 1940s when biguanide was reinvestigated in the search for antimalarial agents and repurposed to treat influenza (93). Another biguanide, which is so-called flumamine, is still in use as an anti-influenza and anti-malarial medication in the Philippines (94). As antivirals, biguanides do not cause lung toxicity. Thus, when inhaled, it may deliver more predictable amount of biguanide to the lung than oral dosing and limit the risk of systemic side-effects (95).

Moreover, metformin exerts anti-inflammatory actions by decreasing the circulating biomarkers of inflammation in people with or without T2DM (96). Metformin can also modulate the immune response and restore immune homeostasis in T cells (97), and more in particular, a reciprocal balance between Th17 and Treg cells *in vitro* and *in vivo* (98). In a cohort of 1,213 hospitalized patients with COVID-19 and pre-existing T2DM, Cheng et al. show that the dynamic trajectories of serum inflammatory factors, including IL-6, IL-2, and TNF-a, all showed lower degrees of elevation in the metformin group than in the non-metformin group, particularly compared to the subgroup of individuals with severe COVID-19 (99). This consists with the findings from Chen et al. that metformin applicants showed overall lower levels of IL-6 on admission compared to ordinary patients (100).

Lately, it was identified that metformin exerts potent antifibrotic effects in the lung by inhibiting transforming growth factor (TGF)-β1 action, suppressing collagen formation, activating peroxisome proliferator-activated receptor γ (PPARγ) signaling and inducing lipogenic differentiation in lung fibroblasts (101). However, there is scant information about the antifibrotic actions of metformin in the context of coronavirus infection.

**Figure 2:**
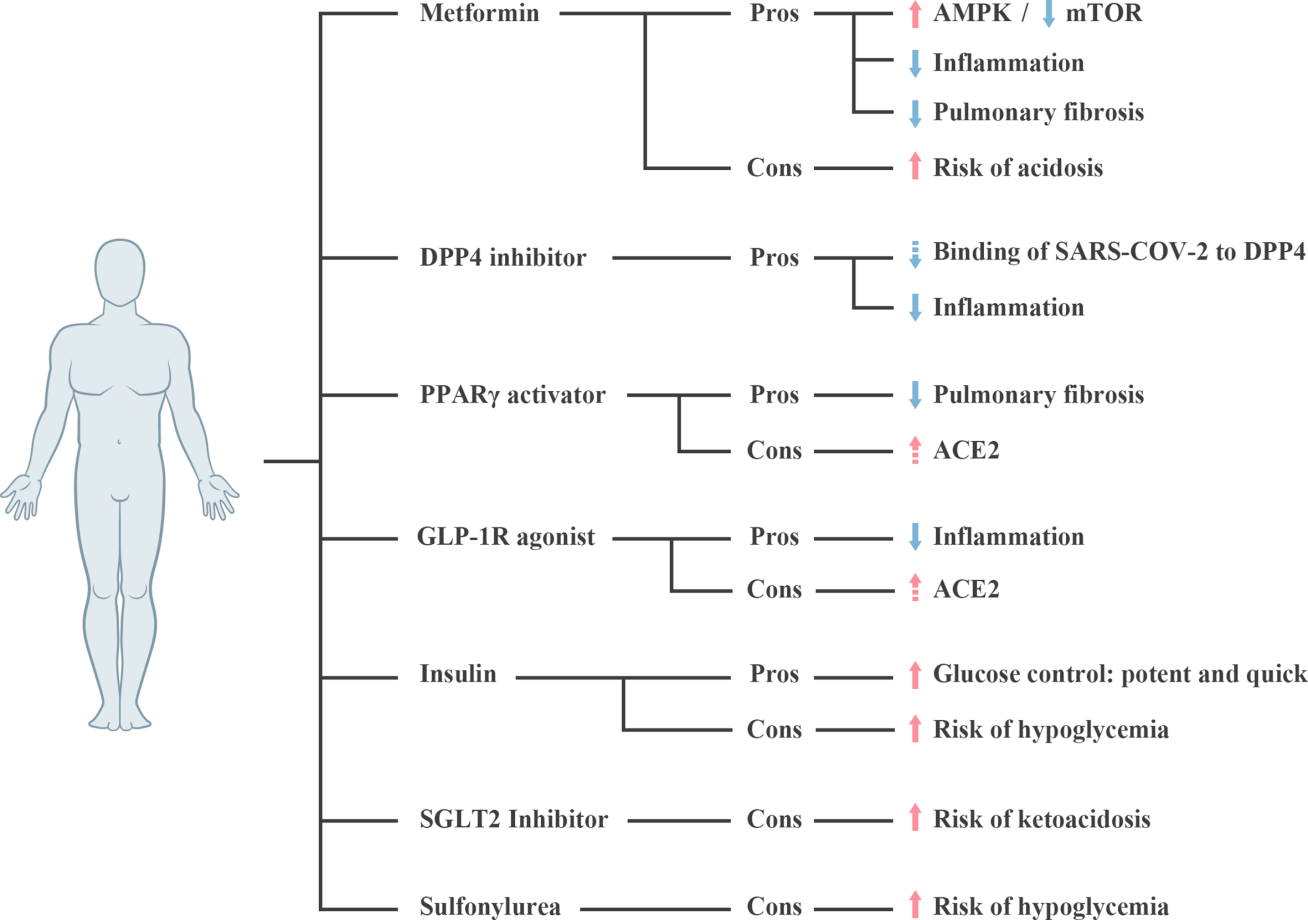
Pros and cons of glycemic lowering agents in diabetic patients with SARS-CoV-2 infection. Dashed arrow represents the assumption requiring further verification. ACE2, Angiotensin-converting enzyme 2; AMPK, Adenosine 5’-monophosphate-activated protein kinase; mTOR, mechanistic target of rapamycin; DPP4, dipeptidyl Peptidase 4; GLP-1R, glucagon-like peptide 1 receptor; PPARγ, peroxisome proliferator-activated receptor γ; SARS-CoV-2, severe acute respiratory syndrome coronavirus 2; SGLT2, sodium-glucose cotransporter 2.

In this respect, metformin may have the potential to benefit diabetic patients with SARS-CoV-2 infection in some ways (**Figure 2**). Interestingly, two observational studies also identified its application resulted in a decrease in the mortality of patients with COVID-19 and T2DM (102, 103). More extensive studies are necessitated to estimate the effectiveness of metformin in COVID-19. Howbeit, metformin-associated acidosis in these patients, particularly in cases with severe COVID19 needs to be noted (99). Regular monitoring of lactic acid levels is recommended after metformin administration in certain patients.

### 5.2 DPP4 Inhibitor

DPP4 inhibitors act selectively to inhibit the catalytic activity of DPP4, and are widely used in clinic for the treatment of T2DM (104). A recent retrospective study identified that the use of DPP4 inhibitors was significantly and independently associated with a lower mortality (Hazard ratio 0.13) and a less severe pneumonia among diabetic patients with SARS-CoV-2 infection (105). Consistent results were reported by Solerte and colleagues, that treatment with sitagliptin, a highly selective DPP4 inhibitor, at the time of hospitalization resulted in an improved clinical outcomes and a greater number of hospital discharges (106).

Interestingly, membrane-associated human DPP4 acts as a functional coronavirus receptor (107). Transgenic mice engineered to express human DPP4 (hDPP4) became susceptible to MERS-CoV (108). Moreover, DPP4 also acts as a functional receptor for hCoV-Erasmus Medical Center (hCoV-EMC), another member of coronavirus family, which was identified to cause severe and sometimes lethal lower respiratory tract infection (107). Furthermore, it was identified that the S1 domain of SARS-CoV-2 spike glycoprotein potentially interacts with the human DPP4 by means of a computational model based selective docking (109).

Although above findings revealed that DPP4 serves as a potential co-receptor for coronaviruses, few data have been shown whether DPP4 inhibitors interfere the binding of SARS-CoV-2 to DPP4. Available evidences were mostly derived from studies on MERS-CoV. Anti-DPP4 antibodies blocked acute MERS-CoV infection in susceptible bat cells in a dose-dependent manner (110). Administration of two human anti-DPP4 antibodies (REGN3051 and REGN3048) could interrupt the interaction between MERS-CoV spike protein and hDPP4, and lead to a mitigated lung pathology in mice with experimental MERS-CoV infection (111). Similarly, Tang et al. also reported that human neutralizing antibodies directed against the receptor-binding domain (RBD) of the MERS-CoV spike protein, which blocked the viral binding to hDPP4 (112).

Our previous review has proposed that DPP4 inhibition also presents potential modulatory functions in the immune system (113), which may contributes to a reduction in inflammatory response in patients with SARS-CoV-2 infection (**Figure 2**). DPP4, originally known as the lymphocyte cell surface protein CD26, is widely expressed in many types of immune cells including CD4^+^ and CD8^+^ T cells, B cells, natural killer (NK) cells, dendritic cells, and macrophages; and plays a role in the regulation of cell function (113). Enzymatic activity inhibition of DPP4 leads to an upregulation of Treg lymphocytes, downregulation of Th17 cells, and suppression on the secretion of pro-inflammatory cytokines, such as IL-1, 6 and 10 (113). Thus, when applied in T2DM patients with SARS-CoV-2 infection, it would be significant to monitor DPP4 inhibitor-induced alterations in immune indexes.

### 5.3 PPARγ Activator and GLP-1R Agonist

Certain anti-diabetic drugs like PPARγ activators have been shown to upregulate ACE2 in animal models (114). Treatment of diabetic rats with liraglutide, a glucagon-like peptide 1 receptor (GLP-1R) agonist, also provoked a strong elevation in pulmonary ACE and ACE2 expression on mRNA levels (115). As such, it is speculated that treatment for diabetes with pioglitazone/liraglutide may increase the risk of severe and fatal COVID-19 development (**Figure 2**).

On the other hand, pulmonary lipofibroblasts located in the alveolar interstitium displayed an ability to differentiate into myofibroblasts, which play an integral role in pulmonary fibrosis (57) and are potentially involved in the exacerbation of pneumonia in patients with SARS-CoV-2 infection. Yet, PPARγ activators are able to stabilize lipofibroblasts in their “inactive” state, preventing the transition of the cells into myofibroblasts, so that they inhibit the development of pulmonary fibrosis (116). The therapeutic effect of rosiglitazone in the murine models of bleomycin-induced pulmonary fibrosis is well determined (117, 118). However, the putative pathophysiological significance of these findings in the context of experimental coronavirus infection has not been fully explored.

GLP-1R agonists exert broad anti-inflammatory actions in humans with T2DM as well as in obese individuals (119). It was revealed that GLP-1 agonists contribute to a reduction in plasma levels of pro-inflammatory cytokines including TNF-α, IL-1β and IL-6, and an increase in plasma levels of adiponectin, which belongs to the anti-inflammatory adipokines, in T2DM patients (120). Moreover, multiple preclinical studies have demonstrated that GLP-1R agonists attenuate pulmonary inflammation and even preserve lung function in rodent models with experimental lung injury (121–124).

So far, the safety about continuous administration of PPARγ activators and GLP-1R agonists in patients with SARS-CoV-2 infection are not stated. Further studies are needed to clarify whether the administration of these two medicines is suitable for diabetic patients with SARS-CoV-2 infection.

### 5.4 Insulin, SGLT2 Inhibitor, and Sulfonylurea

Insulin has been extensively used for decades to control blood glucose in critically ill patients with diabetes (125, 126). Accordingly, insulin therapy has been recommended by many experts for the treatment of diabetic patients with SARS-CoV-2 infection (127, 128). However, in a study with 120 patients with COVID-19 and T2DM by Chen et al., the insulin and non-insulin groups showed no significant difference in the percentages of severe and critical illness on admission (100). Surprisingly, a retrospective analysis of 689 patients with COVID-19 and T2DM revealed a markedly increased mortality in patients with insulin treatment (129). Hypoglycemia may be one of the important drivers causing insulin-associated higher mortality. Accordingly, frequent glucose monitoring, even the application of continuous glucose monitoring may lower the rates of hypoglycemia emergence and improve the clinical outcomes.

Sodium-glucose cotransporter 2 (SGLT2) inhibitors are a novel class of anti-diabetic drugs (OADs) which inhibit the reabsorption of sodium and glucose in urine (130). It has been reported that this kind of OAD could increase ketone accumulation and may induce euglycemic ketoacidosis (euDKA) (130). Besides, SARS-CoV-2 infection may be associated with anorexia, dehydration, and rapid deterioration in clinical status. Although SGLT2 inhibitors are generally well tolerated in the outpatient setting, it may still increase the risk of volume depletion and euDKA in symptomatic individuals with T2DM and acute SARS-CoV-2 infection (131). Accordingly, the use of SGLT2 inhibitors should be cautiously re-evaluated in patients with severe SARS-CoV-2 infection, particularly in patients suffering from dehydration. Furthermore, sulfonylureas should be avoided to use in T2DM patients with gastrointestinal symptoms such as diarrhea and nausea, owing to its hypoglycemia side effect.

### 5.5 Non-Hypoglycemic Agents

ACE inhibitors (ACEIs) and angiotensin II type-I receptor blockers (ARBs) are frequently taken by individuals with diabetes. However, it was found that ACEI and ARB treatment in T1DM and T2DM cause a substantially raise of ACE2 expression in the renal, duodenal, and cardiovascular systems (132–134). Yet, an elevation of ACE2 levels in the respiratory system after ACE/ARBs application has not been reported. Clinical outcomes of patients with SARS-CoV-2 infection who took RAS inhibitors were comparable with those of patients without ACEI/ARB administration (100, 135). Nevertheless, whether these medicines are safe for the treatment of patients with SARS-CoV-2 infection requires more experimental and clinical evidences.

## 6 Conclusions

In this review, the relationship of diabetes and COVID-19 were discussed. However, most of the existing studies failed to distinguish the potential difference of type 1 and type 2 diabetes among COVID-19 subjects. Furthermore, we describe the potential mechanisms which are involved in the regulation of an increased susceptibility and illness severity of diabetic patients to COVID-19. On the other hand, SARS-CoV-2 infection may also trigger the new onset diabetes. The rapid increase in new experimental information stemming from the SARS-CoV-2 epidemic requires careful evaluation to help understand the pathology of COVID-19, especially for the treatment of patients with both diabetes and COVID-19. All medications used in diabetes displayed advantages and disadvantages. Physicians should estimate the status of patients individually, especially the risk of hypoglycemia, acidosis and gastrointestinal symptoms, to optimize the therapeutic strategies for glycemic control in patients with SARS-CoV-2 infection.

## Author Contributions

SS and YC wrote the manuscript. RP and QY edited the manuscript. XY edited the revised manuscript. All authors contributed to the article and approved the submited version.

## Funding

This work was supported by grants from National Natural Science Foundation of China [grant number 82070859 to YC, grant number 81100581 to SS], a grant from Tongji Hospital in HuaZhong University of Science and Technology [grant number 2201103295 to YC], a grant from the Bethune·Merck Diabetes Research Fund [grant number 2018 to SS], and a grant from Cardiac rehabilitation and metabolic therapy research fund [grant number 2018 to SS]. This manuscript has not been published and is not under consideration for publication elsewhere.

## Conflict of Interest

The authors declare that the research was conducted in the absence of any commercial or financial relationships that could be construed as a potential conflict of interest.

## Publisher’s Note

All claims expressed in this article are solely those of the authors and do not necessarily represent those of their affiliated organizations, or those of the publisher, the editors and the reviewers. Any product that may be evaluated in this article, or claim that may be made by its manufacturer, is not guaranteed or endorsed by the publisher.
